# DNA oligonucleotides block viral entry of SARS-CoV-2 Omicron variants

**DOI:** 10.1371/journal.ppat.1014461

**Published:** 2026-07-30

**Authors:** Jorge A. Acuña, Benjamin Gabriel, Kasirajan Ayyanathan, Jesse Miller, Yue Li, Kanupriya Whig, Yongqing Zhu, Marisa McGrath, Peter Hewins, Kellie Ann Jurado, Matthew B. Frieman, David C. Schultz, Sara Cherry

**Affiliations:** 1 Department of Pathology and Laboratory Medicine, Perelman School of Medicine, University of Pennsylvania, Philadelphia, Pennsylvania, United States of America; 2 Department of Biochemistry and Biophysics, Perelman School of Medicine, University of Pennsylvania, Philadelphia, Pennsylvania, United States of America; 3 Center for Pathogen Research, Department of Microbiology and Immunology, University of Maryland School of Medicine, Baltimore, Maryland, United States of America; 4 Department of Microbiology, Perelman School of Medicine, University of Pennsylvania, Philadelphia, Pennsylvania, United States of America; Mayo Clinic, UNITED STATES OF AMERICA

## Abstract

Successful viruses including SARS-CoV-2 antagonize and evade immune detection to establish infection. As population immunity to the ancestral strain has risen, new variants—particularly Omicron—have evolved to avoid detection by both circulating antibodies and the innate immune system. Screening innate immune agonists, we previously identified STING agonists as potent inhibitors of the ancestral variant of SARS-CoV-2. When we explored the sensitivity of Omicron BA.1 to diverse innate stimuli, we found that Omicron is also sensitive to interferons and STING agonists; however, Omicron variants are uniquely restricted by small DNA oligonucleotide ligands known to activate TLR9. Mechanistic studies showed that short, but not long, DNA oligonucleotides of diverse sequences, including those that do not engage TLR9, block Omicron infection at an entry step downstream of TMPRSS2-mediated activation. Using genetic and pharmacological assays we confirmed that activity was independent of TLR9. Furthermore, these oligodeoxynucleotides (ODNs) are active *in vivo*, reducing SARS-CoV-2 Omicron titers. Together, these findings identify a previously unrecognized antiviral activity of short DNA oligonucleotides and establish them as selective inhibitors of SARS-CoV-2 Omicron entry.

## Author summary

SARS-CoV-2 continues to spread in humans by evading innate and adaptive immune detection. Here, we compare the sensitivities of the ancestral WA1 and the Omicron BA.1 variant to a panel of innate immune agonists and find that short DNA oligonucleotides are antiviral against Omicron variants but not earlier strains. Mechanistically we find that ODNs block viral entry independent of innate signaling. Importantly, ODNs can reduce viral loads *in vivo* in mice suggesting a new strategy for therapeutic interventions.

## Introduction

SARS-CoV-2 continues to evolve under immune pressure, giving rise to variants with enhanced transmissibility and immune evasion [1]. Mutations in the Spike protein have been central to this process, enabling variants such as Alpha (B.1.1.7), Delta (B.1.617.2), and especially Omicron (ex. BA.1) to escape antibodies and impact cell-entry mechanisms [2–4]. All SARS-CoV-2 variants bind to the ACE2 receptor and require subsequent Spike cleavage by either TMPRSS2 at the plasma membrane or cathepsins in endosomes, to trigger fusion between the viral and cellular membranes, releasing viral RNA into the cytoplasm [5–8]. The affinity of the Spike protein for ACE2, as well as the expression levels of TMPRSS2 in respiratory epithelial cells plays a major role in establishing infection and influencing transmissibility [9,10]. In addition to evasion of circulating neutralizing antibodies, variant-specific changes in Spike have enhanced its stability, binding, and cleavage, influencing these entry pathways affecting tropism, and disease severity [1,11].

Innate immunity serves as the first barrier to viral infection. Pattern-recognition receptors (PRRs) such as Toll-like receptors (TLRs), RIG-I–like receptors (RLRs), NOD-like receptors (NLRs), and cyclic GMP-AMP synthase (cGAS) detect viral antigens including proteins and nucleic acids to induce transcription of interferons (IFNs) [12–14]. Detection of IFN leads to downstream signaling and the transcription of hundreds of interferon-stimulated genes (ISGs), which limit viral replication through different mechanisms including enhancing viral RNA degradation, blocking viral entry and spread [14,15]. SARS-CoV-2 cytoplasmic sensing is driven primarily through RIG-I and MDA5, yet the virus has evolved numerous strategies to suppress these pathways, such as interfering with PRR sensing and blocking downstream signaling [16–18]. Effective interferon responses, particularly when triggered early, can significantly reduce SARS-CoV-2 replication and prevent severe disease, underscoring the importance of the innate immune system in shaping infection outcomes [18,19]. Successive variants have become more resistant to innate immune control, particularly interferon signaling, giving them a replicative advantage early in infection [20,21].

Endosomal TLRs play a critical role in detecting viral infections by recognizing viral nucleic acids that are taken up into endosomes during viral entry or released from bystander cells during cytolytic infection [22]. Specifically, TLR3 recognizes double-stranded RNA (dsRNA), which is a replication intermediate for many viruses [22]. TLR7 and TLR8 detect single-stranded RNA (ssRNA), which is a characteristic feature of RNA viruses, including coronaviruses [12]. TLR9 senses unmethylated CpG DNA, typically associated with bacteria or DNA viruses, but can also detect mitochondrial DNA released during cell damage caused by infection [23,24]. Once these endosomal TLRs detect viral nucleic acids, they initiate signaling cascades through adaptor proteins like TRIF (for TLR3) or myeloid differentiation primary response gene 88 (MyD88) (for TLR7/8/9), ultimately leading to the production of interferons and pro-inflammatory cytokines [12]. This rapid response helps establish an antiviral state in infected and neighboring cells, while also recruiting other immune cells to the site of infection to help clear the virus. Humans deficient in TLR3 or TLR7 present with severe COVID-19 underscoring their importance in controlling SARS-CoV-2 [25–28]. Moreover, unmethylated CpG DNA ligands targeting TLR9 are utilized as safe and effective vaccine adjuvants [29]. By rapidly detecting infection and coordinating a broad immune response, TLR signaling helps limit viral replication and primes the adaptive immune system to mount a more specific and long-lasting defense.

Despite these established innate immune pathways, the extent to which different SARS-CoV-2 lineages respond to innate immune stimulation remains incompletely understood. Early variants, such as Alpha, already showed enhanced expression of viral proteins, including Orf9b, that block innate immune sensing and signaling pathways, helping the virus replicate more efficiently in the face of interferon responses [20,30]. The Delta and Omicron variants acquired additional mutations that further dampened innate immune detection and interferon activation [21,31]. Some of these adaptations improved the virus’s ability to suppress key pathways involved in sensing viral RNA, such as RIG-I and MDA5 signaling, while others directly interfered with the production or response to interferons [31–33]. Omicron variants show enhanced evasion of RLR-mediated innate immune signaling, however, whether their sensitivity to other PRR-pathway activation may differ from earlier strains is less clear [34]. Understanding these differences is critical, as innate immune agonists remain promising antiviral candidates that could therefore retain activity against highly mutated variants.

Given that Omicron lineages show enhanced innate immune antagonism, we hypothesized that their sensitivity to innate immune agonists differs from that of the ancestral WA1 strain. We screened a panel of diverse innate immune activators that target each of the major PRRs in respiratory epithelial Calu-3 cells against SARS-CoV-2. We found that both ancestral WA1 and Omicron BA.1 were sensitive to interferons, STING agonists, and TLR3 agonists. In contrast, Omicron BA.1 but not WA1 was sensitive to short oligodeoxynucleotides (ODNs) sensed by TLR9. By testing a larger panel of ODNs we found that short, but not long, DNA oligonucleotides of diverse sequences, including those that do not activate TLR9, block infection. Moreover, we found that the antiviral activity of ODNs is independent of TLR9 and canonical TLR signaling. We genetically mapped the activity to Spike as SARS-CoV-2 WA1 expressing the Omicron BA.1 Spike was now sensitive to ODN treatment. This suggests that ODNs block Omicron Spike mediated viral entry. Through mechanistic assays, we found that ODNs do not neutralize virions but instead block infection at a step downstream of TMPRSS2 engagement, implicating an early post-attachment entry step. Finally, treatment with ODN2006 or ODN2395 reduced Omicron BA.1 infection in mice, confirming *in vivo* antiviral activity. Together, these findings identify short DNA oligonucleotides as selective inhibitors of SARS-CoV-2 Omicron entry and reveal a previously unrecognized vulnerability within the Omicron lineage.

## Results

### The DNA oligonucleotide ODN2006 protects cells from SARS-CoV-2 omicron infection

We previously screened a panel of 75 innate immune agonists that activate canonical innate immune signaling pathways for their ability to protect respiratory epithelial cells from infection with the ancestral SARS-CoV-2 strain (WA1) [19]. We identified STING agonists and poly(I:C) as potent antivirals in Calu-3 cells, and these ligands also protected against additional variants, including Beta, both *in vitro* and in mouse models [19,28]. Their antiviral activity required interferon signaling, as treatment with the JAK–STAT inhibitor Ruxolitinib abrogated protection [19].

**Fig 1:**
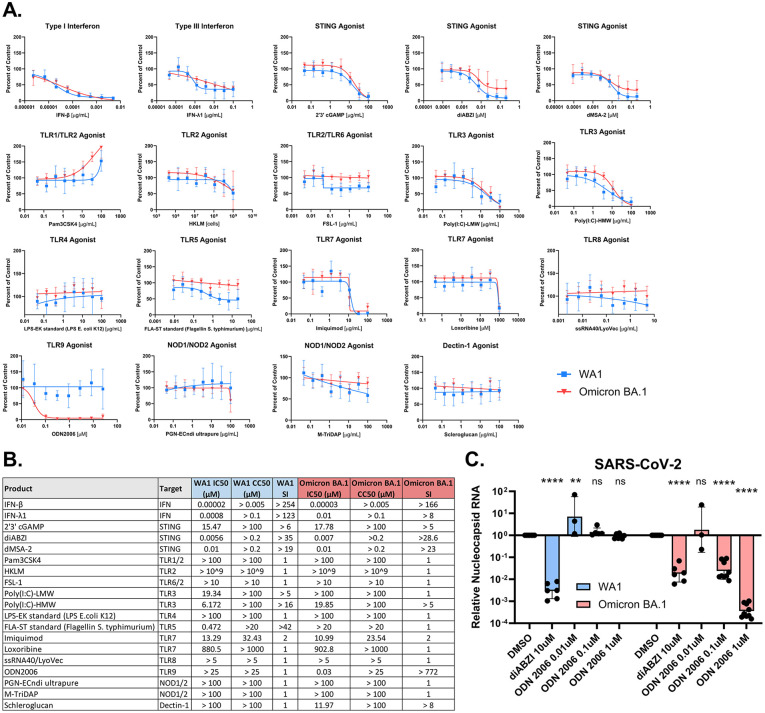
ODN2006 is selectively antiviral against SARS-CoV-2 Omicron BA.1. **(A)** Calu-3 cells were pre-treated with innate immune agonists in 8-pt dose-response for two hours and then infected with SARS-CoV-2 (MOI 0.5) WA1 ancestral strain (blue) or Omicron BA.1 (red) for 48 hours (h) and processed for automated microscopy and image analysis quantifying total cell numbers and percent infection (dsRNA + /nuclei+) to determine Percent-of-Control (POC) % positive. Each data point represents the mean ±SD of n = 6 biological replicates. **(B)** IC50, CC50, and SI are listed for each compound. **(C)** Calu-3 cells were pre-treated with vehicle (DMSO), diABZI positive control, or ODN2006 for 2h and infected with WA1 or BA.1 (MOI 0.2) for 48h prior to processing for RT-qPCR analysis of viral RNA (Nucleocapsid). Means ±SD with individual biological replicates shown. Significance was calculated using One Way ANOVA with Dunnett Correction on DMSO control (**p < 0.01, ****p < 0.0001; ns, no significance).

Given that Omicron variants have now replaced earlier strains globally, we next asked whether Omicron BA.1 exhibits altered sensitivity to innate immune agonists. Calu-3 cells were pre-treated with a panel of agonists in 8-point dose-response format and subsequently infected with either WA1 or Omicron BA.1 for 48 hours before automated imaging. Percent-of-control (POC) infection allowed comparisons across strains. We evaluated Type I (IFN-β) and Type III (IFN-λ1) interferons and three STING agonists: the natural ligand 2′3′-cGAMP, and two non-nucleoside analogs diABZI and dMSA-2 [35,36]. WA1 and BA.1 exhibited comparable sensitivity to all of these treatments, with similar IC₅₀ values and no detectable cytotoxicity (CC₅₀ > 100 μM), although Omicron trended toward modestly decreased sensitivity (Figs 1A, 1B and S1).

We then tested a broader panel of innate immune agonists targeting canonical pattern recognition receptors (PRRs): TLR1/2 (PAM_3_CSK_4_), TLR2 (HKLM), TLR2/6 (FSL-1), TLR3 (Poly(I:C)-LMW, Poly(I:C)-HMW), TLR4 (LPS-EK), TLR5 (FLA-ST), TLR7 (Imiquimod, Loxoribine), TLR8 (ssRNA40/Lyovec), TLR9 (ODN2006), NLRs NOD1/NOD2 (PGN-ECndi, M-TriDAP), and the C-type lectin receptor Dectin-1 (Scleroglucan). The TLR3 agonists exhibited antiviral activity with similar sensitivity between WA1 and BA.1 (Figs 1A, 1B and S1). Most other ligands showed no antiviral activity against either strain, or displayed similar effects on WA1 and BA.1 (Figs 1A, 1B and S1). Unexpectedly, one ligand—ODN2006, a synthetic oligodeoxynucleotide and canonical TLR9 agonist—showed potent antiviral activity specifically against Omicron BA.1 but had no effect on WA1 (Fig 1A). ODN2006 inhibited BA.1 with an IC₅₀ of 30nM and no cytotoxicity (CC₅₀ of >25 μM), yielding a selective index (SI) of 772 (Figs 1B and S1).

To validate this strain-specific activity, we performed an orthogonal assay measuring viral RNA by RT-qPCR of subgenomic (sgRNA) Nucleocapsid RNA. Calu-3 cells were pre-treated with DMSO, the STING agonist diABZI as a positive control, or ODN2006 at 1uM or 0.1uM, before infection with WA1 or BA.1. Consistent with the microscopy assay, diABZI inhibited both strains, whereas ODN2006 selectively inhibited BA.1 replication (Fig 1C).

### Oligodeoxynucleotides selectively inhibit SARS-CoV-2 omicron infection

Synthetic oligodeoxynucleotides (ODNs) are short CpG-containing single-stranded DNA molecules that can function as potent TLR9 ligands depending on their sequence and backbone chemistry [23,37,38]. These unmethylated CpG motifs mimic bacterial DNA and have been developed as vaccine adjuvants because of their ability to activate TLR9 [39–41]. Moreover, CpG ODNs have proven safety profiles as adjuvants in influenza, malaria, cancer neoantigens, and SARS-CoV-2 vaccines, with the FDA-approved ODN CpG 1018 demonstrating improved seroprotection in the Heplisav-B HBV vaccine [42–53]. Stimulatory CpG ODNs fall into three major classes—A, B, and C—based on structural features and their activity on peripheral blood mononuclear cells (PBMCs), particularly B cells and plasmacytoid dendritic cells (pDCs) [38,54–56]. Class A ODNs contain a central palindromic phosphodiester CpG motif with a phosphorothioated (PS) 3’ poly-G tail and strongly induce type I interferons from pDCs [38,57,58]. Class B ODNs have a fully phosphorothioated backbone with linear CpG motifs that activate B cells and NF-κB signaling but weakly stimulate interferon [59–62]. Class C ODNs combine features of both, carrying a PS backbone and palindromic CpG motif that stimulate both B cells and IFN-α production [56]. ODN2006 is a Class B ODN with a full PS backbone and four CpG motifs, giving it high stability and affinity for human TLR9 and making it an effective immune adjuvant [23,57].

**Fig 2:**
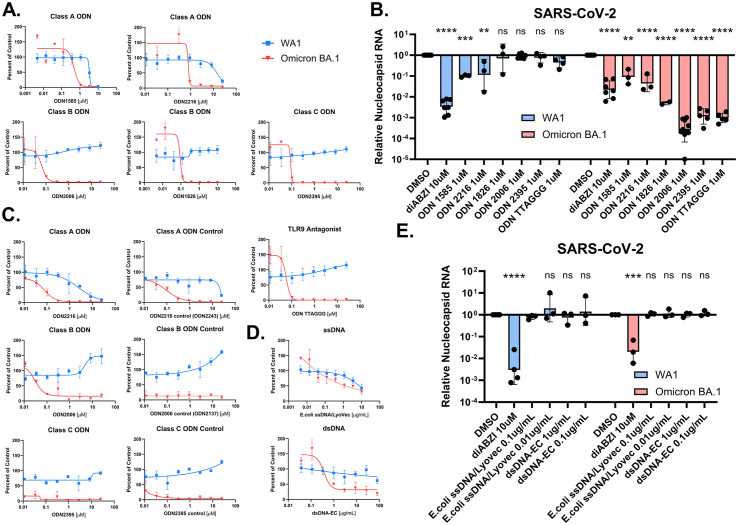
Diverse short oligodeoxynucleotides control SARS-CoV-2 infection. **(A, C, D)** Calu-3 cells were pre-treated with indicated compounds in 8-pt dose-response for 2h and infected with SARS-CoV-2 (MOI 0.5) WA1 ancestral strain (blue) or Omicron BA.1 (red) for 48h and processed for automated microscopy and image analysis quantifying total cell numbers and percent infection (dsRNA + /nuclei+) to quantify Percent-of-Control (POC) % positive. Each data point represents the mean ± SD of n = 2 biological replicates. **(B,E)** Calu-3 cells were pre-treated with vehicle (DMSO), diABZI positive control, or ODNs for 2h and infected with WA1 or BA.1 (MOI 0.2) for 48h and subject to RT-qPCR analysis of viral RNA (Nucleocapsid). Mean±SD with individual biological replicates shown. Significance was calculated using One Way ANOVA with Dunnett Correction on DMSO control (**p < 0.01, ***p < 0.001, ****p < 0.0001; ns, no significance).

Since we found that ODN2006 inhibited Omicron infection we tested additional ODNs from all three classes: Class A ODNs, ODN1585 and ODN2216, which have higher affinity for the mouse and human TLR9 respectively [38,57,58]. We also tested a second Class B ODN, ODN1826, with higher affinity for mouse TLR9 and the Class C ODN, ODN2395 [61,62]. Calu-3 cells were pre-treated with each indicated ODN in dose response and infected with SARS-CoV-2 WA1 or BA.1 for 48 hours. Automated microscopy revealed that ODNs from all three classes inhibited Omicron BA.1, but not WA1 (Figs 2A and S1 and S1 Table). RT-qPCR confirmation demonstrated the selective inhibition of BA.1 replication (Fig 2B). There was modest inhibition of WA1 at high concentrations of ODN1585 and ODN2216 but not the other ligands and not within a physiological range.

To test whether TLR9 activation was required for ODN antiviral activity, we evaluated “control” ODNs that lack CpG motifs and therefore do not activate TLR9: ODN2137 (control for ODN2006), ODN2243 (control for ODN2216), and ODN2395 control [23,63]. Unexpectedly, each of these non-stimulatory ODNs also inhibited BA.1 infection but had no effect on WA1 (Figs 2C and S1 and S1 Table). We further tested ODN TTAGGG, a well-characterized TLR9 antagonist, and found that it likewise selectively blocked BA.1 infection in both microscopy- and RT-qPCR–based assays (Figs 2B, 2C and S1 and S1 Table) [64]. Because all active compounds shared the property of being short DNA oligonucleotides, we asked whether longer DNA molecules could confer similar protection. Treatment with long ssDNA had no antiviral activity against WA1 or BA.1 as measured by microscopy or RT-qPCR (Figs 2D, 2E and S1 and S1 Table). When we tested long dsDNA, there was modest inhibition of BA.1 by microscopy but no inhibition as measured by RT-qPCR (Figs 2D, 2E and S1 and S1 Table). Together, these findings demonstrate that short DNA oligonucleotides, irrespective of CpG content or TLR9 stimulatory capacity, selectively inhibit infection by SARS-CoV-2 Omicron BA.1.

### DNA oligonucleotides inhibit diverse SARS-CoV-2 Omicron variants

**Fig 3:**
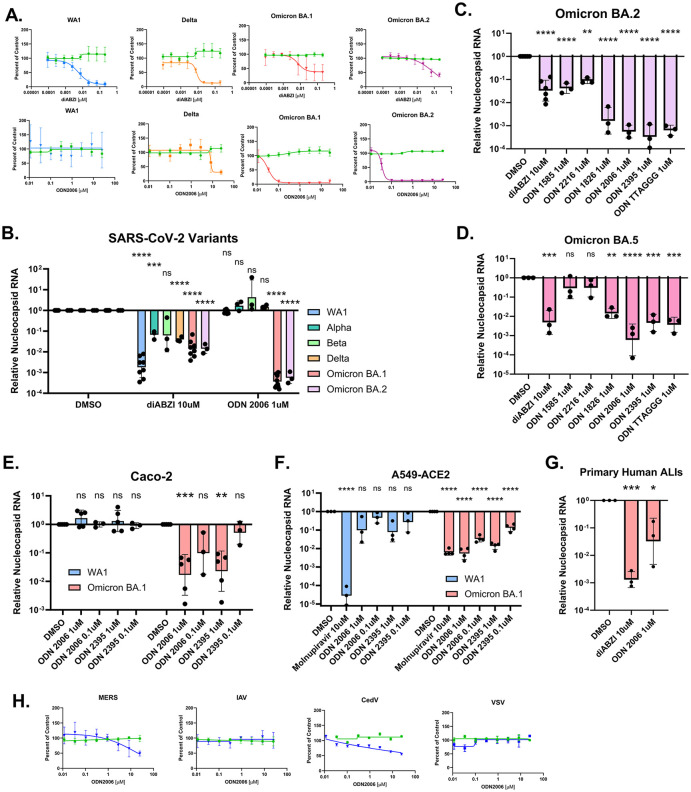
Oligodeoxynucleotides inhibit diverse SARS-CoV-2 Omicron variants. **(A)** Calu-3 cells were pre-treated with indicated agents in 8-pt dose-response for 2h and infected with SARS-CoV-2 variants (MOI 0.5) for 48 hours. Cells were processed for automated microscopy and image analysis quantifying total cell numbers (green) and percent infection (dsRNA + /nuclei+) to determine Percent-of-Control (POC) % positive. Each data point represents the mean±SD n = 3-6 independent biological replicates. **(B, C)** Calu-3 cells were pre-treated with vehicle (DMSO), diABZI positive control, or ODN2006 for two hours and infected with SARS-CoV-2 variants (MOI 0.2) for 48 hours prior to processing for RT-qPCR analysis of viral RNA (Nucleocapsid). **(D)** Calu-3 cells were pre-treated with diABZI positive control or ODNs for 1 hour followed by SARS-CoV-2 Omicron BA.5 (MOI 0.2) infection for 48 hours. Cell lysates were processed for RT-qPCR analysis of viral RNA (Nucleocapsid). **(E)** Caco-2 and **(F)** A549-ACE2 cells were pre-treated with the indicated compounds for 1 hour followed by SARS-CoV-2 WA1 or Omicron BA.1 (MOI 0.2) infection for 48 (Caco-2) or 24 (A549-ACE2) hours. Cell lysates were processed for RT-qPCR analysis of viral RNA (Nucleocapsid). **(G)** Primary human bronchial air-liquid interface (ALI) cells were pre-treated with DMSO vehicle, diABZI positive control, or ODN 2006 1uM for 1 hour followed by SARS-CoV-2 Omicron BA.1 (MOI 0.2) infection for 72 hours. Cell lysates were processed for RT-qPCR analysis of viral RNA (Nucleocapsid). Mean±SD with individual biological replicates shown. Significance for relative viral RNA was calculated using One Way ANOVA with Dunnett Correction for multiple comparisons (*p < 0.05, **p < 0.01, ***p < 0.001, ****p < 0.0001; ns, no significance). **(H)** Calu-3 cells were pre-treated with ODN2006 in 8-pt dose-response for 2h and infected with MERS-CoV (MOI 0.3; 24 hours), IAV (MOI 1; 24 hours), CedV (MOI 0.4; 24 hours), and VSV (MOI 0.05; 24h). Cells were processed for automated microscopy and image analysis quantifying total cell numbers (green) and percent infection (infection + /nuclei+) to determine Percent-of-Control (POC) % positive. Each data point represents the mean±SD n = 2-6 independent biological replicates.

Given the selective activity of ODNs against SARS-CoV-2 BA.1 but not WA1, we next explored whether this antiviral activity extended to additional SARS-CoV-2 variants. We evaluated several early variants of concern, including isolates from lineages B.1.1.7 (Alpha), B.1.351 (Beta), and B.1.617.2 (Delta), as well as a second Omicron lineage, BA.2. Calu-3 cells were pre-treated with DMSO vehicle, the STING agonist diABZI (positive control), or ODN2006 in dose-response prior to infection with each SARS-CoV-2 variant for 48 hours. As expected, diABZI inhibited infection across all variants tested, although Omicron lineages are more resistant (Figs 3A and S1 and S2 Table). In contrast, ODN2006 showed no antiviral activity toward the pre-Omicron variants by either microscopy or RT-qPCR (Figs 3A, 3B and S1 and S2 Table). However, ODN2006 and additional ODNs retained potent antiviral activity against the Omicron BA.2 and BA.5 variants, indicating that the inhibitory effect generalizes across Omicron lineages but does not extend to earlier SARS-CoV-2 strains (Fig 3C and 3D and S2 Table). We next tested whether ODNs were active against pre-Omicron variants at higher concentrations of ODN2006. Calu-3 cells were pre-treated with DMSO, diABZI and Camostat positive controls, or ODN2006 at 10uM prior to infection with SARS-CoV-2 WA1, Delta, or Omicron BA.1 variants. As expected, ODN2006 inhibited Omicron BA.1, but had no activity against WA1 or Delta (S2 Fig).

We next extended our studies to additional cell lines including human intestinal epithelial Caco-2 cells and human respiratory epithelial cells A549 expressing ACE2 (A549-ACE2). Cells were pre-treated with DMSO, Molnupiravir as a positive control, ODN2006, or ODN2395 and infected with SARS-CoV-2 WA1 or Omicron BA.1 for 48 hours. Similar to results in Calu-3 cells, ODN2006 and ODN2395 inhibited Omicron BA.1, but not WA1, across these cell lines (Fig 3E and 3F). Next, we tested ODN activity in primary human bronchial air-liquid interface cells (ALIs) that we previously found are permissive to infection and controlled by IFN and diABZI [19,65]. We pre-treated ALIs with vehicle, diABZI positive control, or ODN2006 and infected with Omicron BA.1 for 72 hours. We observed that diABZI and ODN2006 significantly inhibited Omicron BA.1 infection (Fig 3G).

To determine whether ODNs could inhibit other coronaviruses, we next tested activity against Middle East respiratory syndrome coronavirus (MERS-CoV), a highly pathogenic betacoronavirus. Calu-3 cells were pre-treated with ODN2006 in dose-response and infected with MERS-CoV for 24 hours. ODN2006 displayed no detectable antiviral activity against MERS-CoV (Figs 3H and S1 and S2 Table). Finally, to assess whether ODNs might inhibit viruses from other families, we examined three unrelated RNA viruses: influenza A virus (IAV; Orthomyxoviridae), Cedar virus (CedV; Paramyxoviridae), and vesicular stomatitis virus (VSV; Rhabdoviridae). ODN2006 failed to block infection by any of these viruses (Figs 3H and S1 and S2 Table). Together, these results demonstrate that short DNA oligonucleotides exhibit a striking lineage-specific antiviral effect that appears unique to SARS-CoV-2 Omicron variants and is not observed with earlier SARS-CoV-2 strains, other coronaviruses, or diverse RNA viruses.

### DNA oligonucleotides inhibit Omicron independent of TLR9 and canonical innate signaling

**Fig 4:**
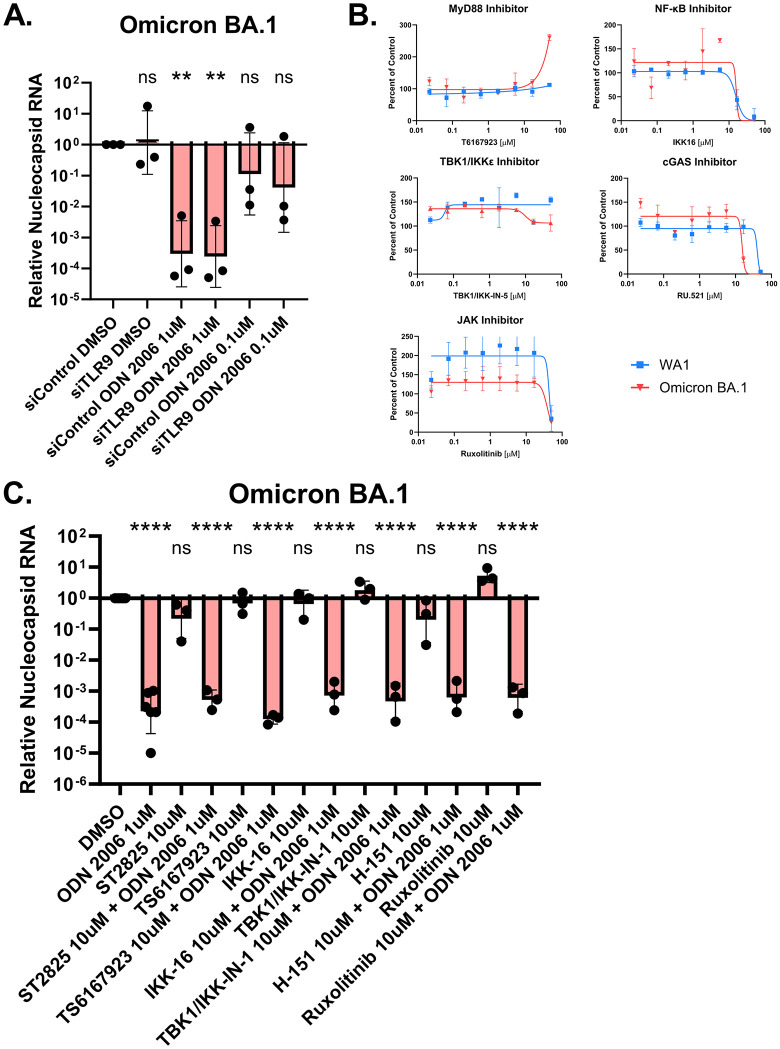
Oligodeoxynucleotides inhibit Omicron infection independent of TLR9 and canonical innate immune signaling. **(A)** Calu-3 cells were transfected with control siRNAs or siRNAs targeting TLR9 and at 48h treated with vehicle (DMSO) or ODN2006 as indicated and subsequently infected with SARS-CoV-2 Omicron BA.1 (MOI 0.2) for 48h prior to processing for RT-qPCR analysis of viral RNA (Nucleocapsid). Mean ±SD with individual biological replicates shown. **(B)** Calu-3 cells were pre-treated with indicated compounds in 8-pt dose-response for 2h and infected with SARS-CoV-2 WA1 (blue) or BA.1 (red) (MOI 0.5) for 48h and processed for automated microscopy and image analysis quantifying total cell numbers and percent infection (dsRNA + /nuclei+) to determine Percent-of-Control (POC) % positive. Each data point represents the mean±SD of n = 3-6 independent biological replicates. **(C)** Calu-3 cells were pre-treated with vehicle (DMSO) or inhibitors for 2h and infected with SARS-CoV-2 Omicron BA.1 (MOI 0.2) and 48hpi processed for RT-qPCR analysis of viral RNA (Nucleocapsid). Mean ±SD with individual biological replicates shown. Significance was calculated using One Way ANOVA with Dunnett Correction on DMSO control (***p < 0.001, ****p < 0.0001; ns, no significance).

TLR9-activating ODNs displayed antiviral activity against SARS-CoV-2 Omicron BA.1, but ODNs that do not engage TLR9 also displayed antiviral activity. These data suggest that the activity would be independent of TLR9. To determine if TLR9 was required for the activity of antiviral ODNs we began by mining transcriptomics data, where we found that Calu-3 cells do not express detectable TLR9 (S3 Table) [19]. Even though the cells did not express detectable levels of TLR9, we tested if siRNA-mediated depletion of TLR9 impacted the activity of antiviral ODNs. Control and TLR9-depleted cells were treated with DMSO or ODN2006 and infected with Omicron BA.1. siRNA-depletion of TLR9 had no effect on infection or on the antiviral activity of ODN2006, indicating that ODN2006 acts independently of TLR9 (Fig 4A).

We next examined whether ODN activity depends on canonical signaling pathways downstream of TLRs and other PRRs. Many TLRs activate NF-κB through MyD88, IRAK, and TRAF6 signaling [22,66,67]. Thus, we compared WA1 and BA.1 sensitivity to the MyD88 inhibitor T6167923, the IKK complex (downstream of IRAK and TRAF6) inhibitor IKK16, and the TBK1/IKKε inhibitor TBK1/IKK-IN-1, finding similar responses for both viruses (Figs 4B and S1 and S4 Table). Because DNA ligands can also activate the cGAS–STING and interferon pathways, we tested the cGAS-STING inhibitor RU.521 and the JAK1/2 inhibitor Ruxolitinib, again observing no differential sensitivity between variants (Figs 4B and S1 and S4 Table). Although, the JAK inhibitor led to higher levels of WA1 infection than BA.1. To determine whether any of these pathways are required for ODN-mediated antiviral activity, we treated Calu-3 cells with inhibitors targeting MyD88 (ST2825), IKK1/2 (IKK-IN-16), TBK1/IKKε (TBK1/IKK-IN-1), STING (H-151), or JAK1/2 (Ruxolitinib), either alone or together with ODN2006, before infection with Omicron BA.1. Only ruxolitinib impacted the baseline infection levels, showing an increase, while the others did not (Fig 4B and 4C). Co-treatment revealed that blocking these pathways did not impact the antiviral effect of ODN2006 (Fig 4C). Together, these results demonstrate that ODN2006 inhibits SARS-CoV-2 Omicron infection through a mechanism that is independent of TLR9, MyD88–NF-κB signaling, cGAS–STING, or JAK–STAT pathways, suggesting a noncanonical antiviral mechanism unique to short DNA oligonucleotides.

### DNA oligonucleotides block Omicron entry

**Fig 5:**
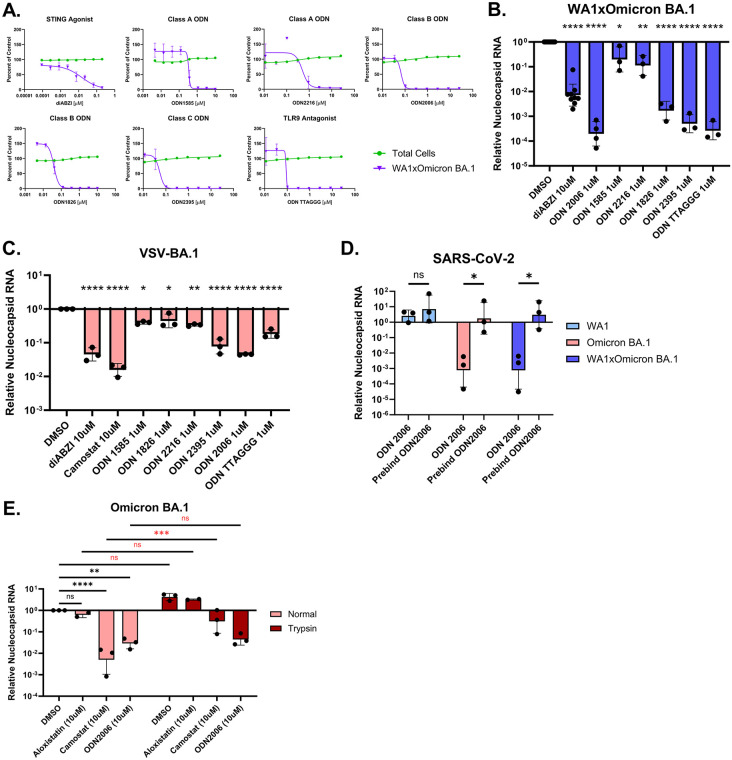
Oligodeoxynucleotides block Omicron entry downstream of TMPRSS2 engagement. **(A)** Calu-3 cells were pre-treated in 8-pt dose-response for 2h and infected with recombinant SARS-CoV-2 WA1xBA.1 (MOI 0.5) for 48h. Cells were processed for automated microscopy and image analysis quantifying total cell numbers and percent infection (dsRNA + /nuclei+) to determine Percent-of-Control (POC) % positive. Each data point represents the mean±SD of n = 2 independent biological replicates. **(B)** Calu-3 cells were pre-treated with vehicle (DMSO) or indicated agents for 2h and infected with recombinant SARS-CoV-2 WA1xBA.1 (MOI 0.2) for 48 hours prior to processing for RT-qPCR analysis of viral RNA (Nucleocapsid). Mean ±SD with individual biological replicates shown. Significance calculated using One Way ANOVA with Dunnett Correction on DMSO control (*p < 0.5, **p < 0.01, ****p < 0.0001). **(C)** Calu-3 cells were pre-treated with diABZI and Camostat positive controls or ODNs for 1 hour followed by VSV-BA.1 (MOI 4) infection for 24 hours. Cell lysates were processed for RT-qPCR analysis of viral RNA (Nucleocapsid). Mean ±SD with individual biological replicates shown. Significance calculated using One Way ANOVA with Dunnett Correction for multiple comparisons (*p < 0.05, **p < 0.01, ***p < 0.001, ****p < 0.0001; ns, no significance). **(D)** SARS-CoV-2 WA1, BA.1, or WA1xBA.1 virions were pre-bound with ODN2006 1uM, and then used to infect Calu-3 cells (MOI 0.2) with the final concentration of ODN2006 at 0.01uM on the cells (‘prebind’). In parallel, cells were directly treated with 1uM ODN2006 and infected with SARS-CoV-2 (MOI 0.2) (‘normal’). 48hpi cells were subject to RT-qPCR analysis of viral RNA (Nucleocapsid). Mean ±SD with individual biological replicates shown. Significance calculated using One Way ANOVA with Dunnett Correction for multiple comparisons (*p < 0.05, **p < 0.01, ***p < 0.001, ****p < 0.0001; ns, no significance). **(E)** Calu-3 cells were pre-treated with indicated drugs for 1h and infected with SARS-CoV-2 (MOI 2) and either treated with TPCK-treated trypsin or media as indicated and 20hpi processed for RT-qPCR analysis of viral RNA (Nucleocapsid). Mean ±SD with individual biological replicates shown. Significance calculated using One Way ANOVA with Sidak Correction for multiple comparisons (**p < 0.01, ***p < 0.001, ****p < 0.0001; ns, no significance).

The most striking difference between Omicron lineages and earlier SARS-CoV-2 variants lies in the Spike protein [68,69]. Omicron BA.1 contains more than 30 amino acid substitutions in Spike relative to WA1—accounting for ~75% of all amino acid differences across the genome [70]. Because Spike mediates attachment and membrane fusion, we hypothesized that ODNs inhibit an early entry step unique to Omicron Spike. To test whether Spike determines ODN sensitivity, we took advantage of a recombinant SARS-CoV-2 WA1 virus encoding the BA.1 Spike (WA1xBA.1) [71]. Calu-3 cells were pre-treated with ODNs and infected with WA1xBA.1. As expected, diABZI robustly inhibited infection (Figs 5A, 5B and S1 and S5 Table). ODNs did not inhibit parental WA1, but replacement of WA1 Spike with BA.1 Spike conferred strong ODN sensitivity, mirroring inhibition of authentic BA.1 (Figs 5A, 5B and S1 and S5 Table). To further confirm that Omicron Spike is required for sensitivity to ODNs, we utilized recombinant Vesicular Stomatitis Virus (VSV) expressing Omicron BA.1 Spike (VSV-BA.1) [72,73]. Calu-3 cells were pre-treated with diABZI and Camostat positive controls, the indicated ODNs, and infected with VSV-BA.1. As expected, diABZI and Camostat inhibited VSV-BA.1 infection (Fig 5C). Importantly, ODNs significantly inhibited infection of VSV-BA.1 (Fig 5C). These data demonstrate that BA.1 Spike is sufficient to confer ODN-mediated inhibition.

We next asked whether ODNs neutralize virions directly, similar to Spike-binding antibodies. We pre-incubated WA1, BA.1, or WA1xBA.1 virions with 1uM ODN2006, the concentration used for treatment of cells, for one hour, and then Calu-3 cells were infected with a 100x dilution of the mix. Thus, the final concentration of ODN2006 added to cells is 0.01uM, below the active concentration. As a control for ODN activity, we pre-treated Calu-3 with 1uM ODN2006 and infected with the same MOI as the prebind condition. We also included buffer and Spike neutralizing antibody (Sotrovimab) controls for neutralization of virions. As expected, Sotrovimab inhibited infection of all SARS-CoV-2 viruses during prebind and normal treatments (S3 Fig). ODN2006 had no effect on WA1 either during prebind or on cells (Fig 5D). In contrast, treatment of cells with 1uM ODN2006 inhibited BA.1 and WA1xBA.1, while pre-binding of 1uM ODN2006 had no effect (Fig 5D). Thus, ODN2006 does not act through direct virion neutralization.

This led us to perform an additional assay to define the step of entry inhibited by ODNs. We used a biochemical bypass assay that can determine whether an inhibitor functions upstream or downstream of the Spike cleavage step [74]. We previously showed that SARS-CoV-2 infection of Calu-3 cells is dependent on the surface protease TMPRSS2 and independent of endosomal cathepsins [7]. Therefore, TMPRSS2 inhibition by Camostat blocks Spike protein cleavage required for entry; and this can be bypassed with exogenous addition of trypsin to cleave Spike [74]. Compounds that are bypassed by the addition of trypsin function either at or before Spike cleavage during SARS-CoV-2 entry while compounds that are not bypassed function downstream of Spike cleavage. Calu-3 cells were treated with vehicle, the TMPRSS2 inhibitor Camostat, the cathepsin inhibitor Aloxistatin, or ODN2006, prior to binding of SARS-CoV-2 Omicron BA.1. After an hour, any unbound virus was washed off, and cells were either incubated with TPCK-treated trypsin to promote Spike cleavage and viral entry, or incubated with media, at 37°C for five minutes. Cells were then washed and incubated with the respective compounds for 20 hours and viral infection quantified by RT-qPCR. As expected, Camostat and ODN2006, but not Aloxistatin, blocked BA.1 infection under normal conditions (Fig 5E). Upon trypsin treatment, Camostat lost activity while ODN2006 remained fully inhibitory (Fig 5E). Since Camostat can be bypassed but not ODN2006, these data suggest that ODN2006 acts downstream of TMPRSS2-dependent cleavage to promote viral entry.

### DNA oligonucleotides attenuate Omicron infection *in vivo*

**Fig 6:**
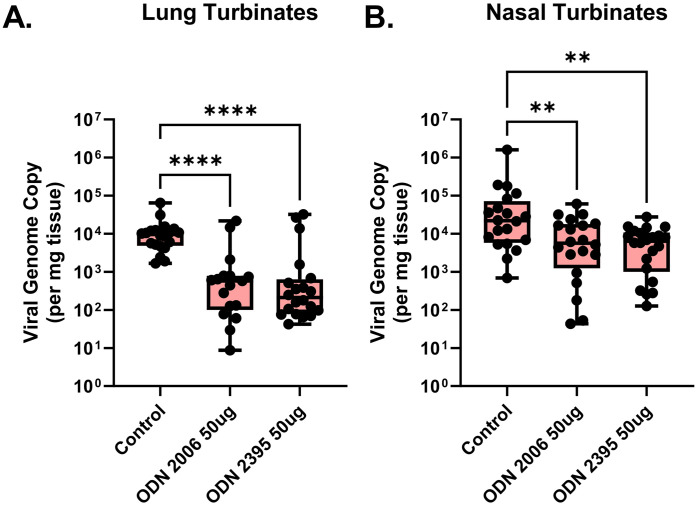
Oligodeoxynucleotides attenuate Omicron infection in vivo. BALB/c mice were treated intranasally with vehicle control (PBS), 50µg of ODN2006, or 50µg of ODN2395, and 24h later intranasally inoculated with 10^4^ PFU of SARS-CoV-2 Omicron BA.1. 48hpi viral RNA was quantified by RT-qPCR (Nucleocapsid) in the **(A)** lung and **(B)** nasal turbinates. Mean ±SD with individual mice shown. Significance for relative viral RNA was calculated using One Way ANOVA with Dunnett Correction on DMSO control (**p < 0.01,****p < 0.0001).

Given the strong Omicron-specific antiviral activity of *ODNs in vitro*, we next tested whether ODNs could reduce viral replication *in vivo*. We tested ODN2006 and ODN2395 which have been used in mice for immunostimulatory studies [56,75–78]. BALB/c mice were treated intranasally with vehicle control (PBS) or 50 µg of ODN2006 or 50 µg ODN2395, and 6 hours later were intranasally inoculated with 10^4^ PFU of SARS-CoV-2 Omicron BA.1 as we have previously shown that this dose leads to infection that peaks at Day 2 [19]. Viral loads were quantified by RT-qPCR in lungs and nasal turbinates at Day 2 post infection. Vehicle treated mice had the highest viral burdens, with median titers of 8,740 copies/mg in lung tissue and 25,391 copies/mg in nasal turbinates (Fig 6A and 6B).

Both ODNs reduced viral loads, with stronger effects in the lung than the upper airway. ODN2006-treated mice showed reduced titers of 442 copies/mg in lungs and 13,028 copies/mg in nasal turbinates. ODN2395 showed the greatest antiviral activity in both tissues, with median titers of 159 copies/mg in lungs and 8,046 copies/mg in nasal turbinates. Together, these data indicate that DNA oligonucleotides can attenuate SARS-CoV-2 Omicron BA.1 infection *in vivo*, with the strongest effects observed in lungs.

## Discussion

We compared the activity of a broad panel of innate immune agonists against the ancestral SARS-CoV-2 WA1 strain and the Omicron BA.1 variant to determine whether these lineages differ in their sensitivity to innate immune activation. Although both viruses responded similarly to interferons, STING agonists, and polyI:C, we identified the TLR9 agonist ODN2006—a synthetic short single-stranded oligodeoxynucleotide—as a potent inhibitor of Omicron BA.1 but not WA1. This striking lineage specificity prompted us to define the mechanism underlying the selective inhibition of Omicron by ODN2006. We found that diverse ODNs that activate TLR9 block Omicron; however, when we tested ODNs that do not activate TLR9, all ODNs selectively blocked BA.1, suggesting that TLR9 signaling is not required. We further found that longer ssDNA or dsDNA ligands were inactive, indicating that ODN antiviral activity is length-dependent. Furthermore, ODN2006 remained fully active in the presence of innate immune pathway-specific inhibitors that disrupt canonical TLR-dependent signaling, further supporting a TLR- and innate immune-independent mechanism of action.

To define the breadth of this antiviral activity, we evaluated ODN activity against additional SARS-CoV-2 variants including Alpha, Beta, Delta, Omicron BA.2, Omicron BA.5, as well as the distantly related MERS-CoV and unrelated RNA viruses including IAV, CedV, and VSV. ODNs selectively inhibited Omicron variants, including BA.2 and BA.5, but showed no activity against pre-Omicron SARS-CoV-2 lineages or other viruses. Because Omicron lineages differ most substantially from earlier variants in the Spike protein, we hypothesized that ODNs target an Omicron-specific feature of Spike and thereby interfere with viral entry. Supporting this model, ODNs inhibited infection of a recombinant SARS-CoV-2 WA1 virus expressing the Omicron BA.1 Spike (WA1 × BA.1) as well as a recombinant VSV expressing BA.1 Spike (VSV-BA.1). Virus neutralization and trypsin-bypass experiments revealed that ODN2006 does not neutralize virions and acts downstream of Spike cleavage, indicating disruption of a late entry step. Finally, we tested whether ODNs protect against Omicron infection *in vivo* due to their implementation as safe and effective vaccine adjuvants [42–50,52]. Intranasal treatment with two different ODNs markedly reduced viral loads, demonstrating that ODNs can attenuate Omicron infection of respiratory tissues.

Taken together, our data indicate that ODNs inhibit SARS-CoV-2 Omicron variants through a mechanism that is independent of canonical innate immune signaling and instead targets a late step of viral entry mediated by the Omicron Spike protein. Entry is a highly vulnerable stage of the viral life cycle, and numerous therapeutics act by blocking viral attachment, proteolytic activation, or membrane fusion [79–82]. Furthermore, we found that ODNs inhibit SARS-CoV-2 Omicron in Caco-2 and A549-ACE2 cells as well as primary ALIs. Because ODNs are active in cells that utilize TMPRSS2-dependent plasma-membrane fusion and cathepsin-dependent entry these data suggest that ODNs likely interfere with a post-fusion step, likely in an early endosome [6,7]. Indeed, ODNs are known to traffic to endosomes for engagement with TLR9 [83–86]. The strict specificity for Omicron suggests that this mechanism exploits an evolutionary divergence in Spike trafficking or engagements with cellular factors. Future studies will be essential to define the molecular interface between ODNs and Omicron Spike and to determine whether this lineage-specific vulnerability can be leveraged for the development of novel antiviral strategies.

## Materials and methods

### Cells and viruses

Calu-3 cells (American Type Culture Collection, ATCC, HTB-55) were cultured in minimum essential medium supplemented with 10% (v/v) fetal bovine serum, 1% (v/v) non-essential amino acids, 1% (v/v) penicillin/streptomycin, and 1% (v/v) Gltuamax (Invitrogen) at 37°C, and 5% CO2. Caco-2 cells (ATCC, HTB-37) were cultured in MEM alpha supplemented with 20% (v/v) fetal bovine serum, 1% (v/v) penicillin–streptomycin and 1% (v/v) l-glutamine. A549-ACE2 cells were cultured in RPMI-1640, 10% fetal bovine serum (FBS), 1% penicillin/streptomycin, and 1% Glutamax. Primary human bronchial air liquid interface cells (ALIs) were obtained from MatTek (AIR-100) and used after day 30. Tissues were maintained and fed twice a week until use [19,65].

SARS-CoV-2 isolates were obtained from BEI Resources; USA WA1/2020 strain (Cat# NR-52281), Alpha Isolate hCoV-19/England/204820464/2020 lineage B.1.1.7 (Cat# NR-54971), Beta Isolate hCoV-19/USA/MD-HP01542/2021 Lineage B.1.351 (Cat# NR-55282), Delta Isolate hCoV-19/USA/MD-HP05285/2021, Omicron BA.1 Isolate hCoV-19/USA/MD-HP20874/2021 Lineage B.1.1.529 (Cat# NR-56461), Omicron BA.2 Isolate hCoV-19/USA/MD-HP49081/2023 (Lineage DV.7.1) in VTA Cells (Cat# NR-59702), and Omicron BA.5 Isolate hCoV-19/USA/COR-22–063113/2022 (Lineage BA.5; Omicron Variant) (Cat# NR-58616). SARS-CoV-2 USA WA1/2020 expressing SARS-CoV-2 Omicron BA.1 Spike (WA1xOmicron BA.1 Spike) was provided by Matthew Frieman. Stocks were prepared by infection of Vero-TMPRSS2 cells in DMEM supplemented with 2% (v/v) fetal bovine serum, 1% (v/v) penicillin-streptomycin, 1% (v/v) L-glutamine and 10 mM HEPES for 3 days, freeze-thawed and clarified by centrifugation (P0). Viral stock titers were determined by tissue culture infectious dose (TCID_50_) using the Reed-Meunch method in Vero-TMPRSS2 cells [7]. Seed stocks were sequence verified, amplified in Vero-TMPRSS2 (P1) and used for all experiments. rMERS-CoV was a gift from Ralph Baric. All work with SARS-CoV-2 and MERS-CoV was performed in a Biosafety Level 3 laboratory and approved by the Institutional Biosafety Committee and Environmental Health and Safety. Vesicular stomatitis virus (VSV-GFP) and VSV expressing Omicron BA.1 Spike (VSV-BA.1) were a gift from Sean Whelan Washington University. Cedar virus (CedV-GFP) was a gift from Christopher Broder USAMRIID. Influenza A virus (IAV PR8) was a gift from Scott Hensley, University of Pennsylvania.

### Dose responses

Compounds, proteins, and microbial ligands are listed in S5 Table. Automated microscopy experiments were performed as described previously [7]. Calu-3 cells (8,000 cells per well) were seeded in collagen-coated 384-well plates (Corning BioCoat). The next day, compounds were added in an eight-point dose-response with 3-fold dilutions between concentrations. The positive control (10 uM remdesivir, n = 32) and the negative control [0.2% dimethyl sulfoxide (DMSO), n = 32] were spotted on each assay plate. Two hours after addition of compounds, cells were infected with SARS-CoV-2 at a multiplicity of infection (MOI) of 0.5. Cells were fixed 48 hours post-infection (hpi) in 4% formaldehyde/phosphate-buffered saline (PBS) for 15 min at room temperature and then washed three times with PBS. Cells were blocked with 2% bovine serum albumin (BSA)/0.1% Triton X-100 in PBS (PBST) for 60 min and incubated with anti-dsRNA (mAbJ2) or anti-SPIKE (Sotrovimab) antibody overnight at 4°C. Cells were washed 3 × in PBST and incubated in secondary antibody (anti-mouse or -human Alexa 488) and Hoechst 33342 for one hour at room temperature. Cells were washed 3 × in PBST and imaged at 10 × using ImageXpress Micro XLS (Molecular Devices) capturing four sites per well. The total number of cells and number of dsRNA/SPIKE-positive cells were measured using the cell scoring module (MetaXpress 6.5.5), and the percentage of infected cells was calculated. Sample well infection was normalized to aggregated DMSO plate control wells and expressed as percentage of control (POC) and Z score in Spotfire (Revvity). A non-linear regression curve fit (GraphPad Prism 10) was performed on the POC of percent infection and cell viability using log10-transformed concentration values to calculate EC50 values for percent infection and CC50 values for cell viability for each drug–cell line combination. The EC50 and CC50 values represent the average of two or more independent experimental replicates. Selective Index (SI) was calculated as the ratio of CC50 and EC50 values (SI = CC50/ EC50) of a drug. Error bars in the dose-response curves represent the SD of replicate data for each drug concentration tested in independent experiments.

### RNA isolation and RT-qPCR

Calu-3 (7 × 10^5^ cells per well) were seeded into collagen coated six-well plates. The next day, if indicated, cells were pretreated with compounds for 1 hour at 37°C. Cells were inoculated with SARS-CoV-2 at the indicated MOI for the indicated time. Total RNA was isolated using TRIzol reagent (Invitrogen) and purified using RNA Clean and Concentrator Kits (Zymo Research). Complementary DNA was synthesized using 1 µg total RNA, Moloney murine leukemia virus (M-MLV) Reverse Transcriptase (Invitrogen), and Random Primers (Invitrogen). Gene-specific primers and Power SYBR Green PCR Master Mix (Applied Biosystems) were used to amplify cellular and viral RNA using the QuantStudio 6 Flex Real-Time PCR Systems (Applied Biosystems). The relative expression levels of target genes were calculated using the standard curve method and normalized to 18S ribosomal RNA as an internal control [7,19]. Primers used in this study are shown in S6 Table.

### siRNA transfections

Gene knockdown was performed using two different siRNAs targeting the TLR9 gene (S7 Table). Cells were transfected with 25nM siRNA using Lipofectamine RNAiMax reagent (Thermo Scientific) according to the manufacturer’s protocol. Calu-3 cells were incubated in the presence of siRNAs for 16–20 hours, followed by a single media change. At 48 hours after the initiation of transfection, cells were treated with the indicated compounds and incubated for one hour before virus addition. Cells were harvested at 48 hours post infection.

### Neutralization assay

To test neutralization, Opti-MEM buffer (Thermo Fisher Scientific), Spike Antibody (Sotrovimab 7,600ng/mL), or ODN2006 (1uM) and virus were incubated for 1 hour at 37°C. After the initial incubation, the virus-compound solutions were added to Calu-3 cells at a 100X dilution such that the final concentration of ODN2006 was 0.01uM. In parallel, Opti-MEM buffer, Sotrovimab, or ODN2006 (1uM) was added to cells and virus was added to the drug-treated cells. The cells were harvested at 48 hours post infection.

### SARS-CoV-2 trypsin bypass

Calu-3 cells were treated with the indicated compounds for two hours prior to SARS-CoV-2 (MOI 2) inoculation. SARS-CoV-2 was bound to cells at 4°C for 30 minutes followed by a cold PBS wash. Following binding, either 10µg/mL of TPCK-treated trypsin (ThermoFisher) or media was added, and plates were incubated for five minutes at 37°C to enable synchronized entry. Cells were then washed with PBS and fresh media was added with fresh compounds. Cell lysates were collected 20 hours post infection.

### Mouse experiments and measurement of viral burden

Animal studies were carried out in accordance with the recommendations in the Guide for the Care and Use of Laboratory Animals of the National Institutes of Health. All animal studies were done with approval by the Institutional Animal Care and Use Committee at the University of Pennsylvania Animal BSL3 (RRID:SCR_022372). Virus inoculations were performed under anesthesia that was induced and maintained with ketamine hydrochloride and xylazine. All efforts were made to minimize animal suffering. BALB/c mice were obtained from the Jackson Laboratory and housed in groups on a standard chow diet. Mice of both sexes were treated with 50nL of PBS, 50µg of ODN2006, or 50µg of ODN2395 and six hours later the animals were inoculated with 10^4^ plaque forming units (PFU) of SARS-CoV-2 Omicron BA.1 intranasally. All mice were euthanized at 48 hours post-infection. Lungs and nasal turbinates were harvested two days after infection and weighed. Total RNA was subjected to viral RNA quantification by RT-qPCR. Primers used are listed in S6 Table.

### Statistical analysis

Statistical analyses were performed using GraphPad Prism 10 Software. Statistical significances were conducted using one-way ANOVA with corrections for multiple comparisons. Adjusted *p* values are described by asterisks in figures: (*) for p < 0.05, (**) for p < 0.01, (***) for p < 0.001, and (****) for p < 0.0001. Relative values with compounds are normalized to DMSO control, siRNA transfections are normalized to non-targeting siRNA control, and mouse infections are normalized to PBS control. Reanalysis of RNAseq data was conducted using raw Calu-3 fastq files that were trimmed, counted, and aligned as previously described [19]. Transcript counts were collapsed to the gene level in R using tximport v1.34.0 and transcripts per million (TPM) count values were determined.

## Supporting information

S1 Fig Compound cytotoxicity. Calu-3 cells were pre-treated with the indicated compounds in 8-pt dose-response for two hours and then infected with SARS-CoV-2 (MOI 0.5) WA1 ancestral strain (blue) or Omicron BA.1 (red) for 48 hours and processed for automated microscopy and image analysis quantifying total cell numbers. Each data point represents the mean ±SD of n = 6 biological replicates.
(TIF)

S2 Fig Higher concentrations of ODN2006 do not inhibit WA1 or Delta variants. Calu-3 cells were pre-treated with diABZI and Camostat positive controls or ODN 2006 10uM for 1 hour followed by SARS-CoV-2 WA1, Delta, or Omicron BA.1 (MOI 0.2) infection for 48 hours. Cell lysates were processed for RT-qPCR analysis of viral RNA (Nucleocapsid). Means + SD for individual biological replicates are shown. Significance for relative viral RNA was calculated using One Way ANOVA with Dunnett Correction for multiple comparisons (*p < 0.05, **p < 0.01, ***p < 0.001, ****p < 0.0001; ns, no significance).
(TIF)

S3 Fig Sotrovimab neutralizes SARS-CoV-2 variants. SARS-CoV-2 WA1, BA.1, or WA1xBA.1 virions were pre-bound with buffer (Opti-MEM) or antibody (Sotrovimab) controls and then used to infect Calu-3 cells at (MOI 0.2) (‘prebind’). In parallel, cells were directly treated with buffer (Opti-MEM) or antibody (Sotrovimab) controls and infected with SARS-CoV-2 (MOI 0.2) (‘normal’). 48hpi cells were subject to RT-qPCR analysis of viral RNA (Nucleocapsid). Mean ±SD with individual biological replicates shown.
(TIF)

S1 Table Anti-viral activity of ODNs against SARS-CoV-2 WA1 and Omicron BA.1. Calu-3 cells were pre-treated with the indicated DNA ligands in 8-point dose response and infected with SARS-CoV-2 WA1 or Omicron BA.1 (48 hours; MOI 0.5), followed by automated microscopy and image analysis quantifying total cell numbers and percent infection. Each compound’s IC50, CC50, and SI is listed for each virus. Data are presented as mean values of n = 2 independent biological replicates.
(XLSX)

S2 Table Anti-viral activity of ODN 2006 against SARS-CoV-2 variants and additional viruses. Calu-3 cells were pre-treated with the indicated ODNs in 8-point dose response and infected with the indicated viruses followed by automated microscopy and image analysis quantifying total cell numbers and percent infection. Each compound’s IC50, CC50, and SI is listed for each virus. Data are presented as mean values of n = 2 independent biological replicates.
(XLSX)

S3 Table Calu-3 TLR RNAseq TPM counts. Previously published Calu-3 RNAseq data was re-analyzed for transcripts per million (TPM) count values for TLR expression across three replicates [19].
(XLSX)

S4 Table Innate Immune Pathway Inhibitor antiviral activity. Calu-3 cells were pre-treated with the indicated innate immunity pathway inhibitors in 8-point dose response and infected with SARS-CoV-2 WA1 or Omicron BA.1 (48 hours; MOI 0.5), followed by automated microscopy and image analysis quantifying total cell numbers and percent infection. Each compound’s IC50, CC50, and SI is listed for each virus. Data are presented as mean values of n = 2 independent biological replicates.
(XLSX)

S5 Table Anti-viral activity of ODNs against SARS-CoV-2 WA1xOmicron BA.1. Calu-3 cells were pre-treated with the indicated ODNs in 8-point dose response and infected with WA1xOmicron BA.1 followed by automated microscopy and image analysis quantifying total cell numbers and percent infection. Each compound’s IC50, CC50, and SI is listed. Data are presented as mean values of n = 2 independent biological replicates.
(XLSX)

S6 Table Compounds used. (XLSX)

S7 Table Primer sequences for RT-qPCR. (XLSX)

S8 Table TLR9 siRNA sequences. (XLSX)
